# A novel 3D bilayer hydrogel tri-culture system for studying functional motor units

**DOI:** 10.1186/s13578-023-01115-2

**Published:** 2023-09-12

**Authors:** Yu-Lung Lin, Jennifer Nhieu, Thomas Lerdall, Liming Milbauer, Chin-Wen Wei, Dong Jun Lee, Sang-Hyun Oh, Stanley Thayer, Li-Na Wei

**Affiliations:** 1 Department of Pharmacology, University of Minnesota Medical School, Minneapolis, MN 55455 USA; 2 Department of Electrical and Computer Engineering, University of Minnesota, Minneapolis, MN 55455 USA; 3 The Ph.D. Program for Translational Medicine, College of Medical Sciences and Technology, Taipei Medical University, Taipei, Taiwan; 4 International Ph.D. Program for Translational Science, College of Medical Science and Technology, Taipei Medical University, Taipei, Taiwan

**Keywords:** Motor unit, Motor neuron, Muscle, Schwann cell, Neuromuscular, Hydrogel, Co-culture, Motor neuron disorders

## Abstract

**Background:**

A motor unit (MU) is formed by a single alpha motor neuron (MN) and the muscle fibers it innervates. The MU is essential for all voluntary movements. Functional deficits in the MU result in neuromuscular disorders (NMDs). The pathological mechanisms underlying most NMDs remain poorly understood, in part due to the lack of in vitro models that can comprehensively recapitulate multistage intercellular interactions and physiological function of the MU.

**Results:**

We have designed a novel three-dimensional (3D) bilayer hydrogel tri-culture system where architecturally organized MUs can form in vitro. A sequential co-culture procedure using the three cell types of a MU, MN, myoblast, and Schwann cell was designed to construct a co-differentiating tri-culture on a bilayer hydrogel matrix. We utilized a µ-molded hydrogel with an additional Matrigel layer to form the bilayer hydrogel device. The µ-molded hydrogel layer provides the topological cues for myoblast differentiation. The Matrigel layer, with embedded Schwann cells, not only separates the MNs from myoblasts but also provides a proper micro-environment for MU development. The completed model shows key MU features including an organized MU structure, myelinated nerves, aligned myotubes innervated on clustered neuromuscular junctions (NMJs), MN-driven myotube contractions, and increases in cytosolic Ca^2+^ upon stimulation.

**Conclusions:**

This organized and functional in vitro MU model provides an opportunity to study pathological events involved in NMDs and peripheral neuropathies, and can serve as a platform for physiological and pharmacological studies such as modeling and drug screening. Technically, the rational of this 3D bilayer hydrogel co-culture system exploits multiple distinct properties of hydrogels, facilitating effective and efficient co-culturing of diverse cell types for tissue engineering.

**Supplementary Information:**

The online version contains supplementary material available at 10.1186/s13578-023-01115-2.

## Background

The Neuromuscular system includes motor neurons (MNs) and their innervated skeletal muscles [1]. The upper MNs in the motor cortex project to the lower (spinal) MNs which then send signals to the innervated muscles to induce muscle contraction. The motor unit are defined as the single alpha motor neuron and the muscle fibers it innervates [2]. The axons of MNs are myelinated by Schwann cells. Neuromuscular junctions (NMJs) are highly specialized synapses that form between the MN axon terminal and myofibers [3]. Physiologically, axon terminals release acetylcholine (ACh) to stimulate post-synaptic ACh receptors (AChR) on myotubes to induce contraction. This neuromuscular system controls all voluntary movements including essential activities like breathing. Deficits in MNs, skeletal muscles, or NMJs lead to neuromuscular disorders (NMDs) that span a spectrum of diseases [4–6] such as amyotrophic lateral sclerosis, myopathy, muscular dystrophy, myasthenia gravis, and age-related sarcopenia [6]. The pathological mechanisms underlying most NMDs remain unclear [4, 7]. Furthermore, there is still no cure for most NMDs. Only two FDA approved drugs, Riluzole and Edaravon, are available for ALS treatment; but their clinical efficacy is not satisfactory [8, 9]. Although there are already many in vitro neuromuscular junction models, a novel model specifically designed for studying MUs is still needed. [7, 8, 10].

In the last two decades, novel bioengineering and cellular technologies have advanced the development of in vitro NMJ models [11–13]. In particular, progress in culture technologies, biomaterials, and microfluidic devices has facilitated the advancement from two-dimensional cultures to multicellular three-dimensional (3D) model systems [13]. The MNs and skeletal muscles used in these previous in vitro models were typically obtained from dissociated explants, primary cells, commercial cell lines, embryonic stem cells (ESCs) [14], and induced pluripotent stem cells (iPSCs) [15, 16], etc. For example, a ‘NMJ chip’ assembled within a microfluidic device was established using human iPSCs [16] for drug screening and precision medicine application in neuromuscular diseases. This procedure takes about 2–3 months to complete and requires advanced equipment. A NMJ model with cultured 3D skeletal muscle tissues was designed to study adult human NMJ development and neuromuscular diseases [17]. A 3D human trunk neuromuscular organoids system was established and shown to exhibit functional NMJ features [18]. However, these systems were established mostly for certain specific objectives [13] and are relatively time- and effort-intensive. A more generalized, functional NMJ model that can be used for a wide spectrum of applications is still needed.

In this current study, we report a novel 3D NMJ system, generated by optimizing a bilayer hydrogel to support organized co-differentiation/maturation of three essential cell types of the NMJ (tri-culture) into in vitro functional MUs with topologically organized 3D structure of NMJs. There are several advantages in this newly developed in vitro NMJ model. First, MN cells are seeded at a low density, allowing every single MN, MN’s axon and axon terminal, as well as the innervated myotube in the MU to be examined. Secondly, the generated MUs are functional and contain Schwann cells which not only are important for axon and NMJ formation and maintenance [19, 20] but also are frequently related to disease conditions [21, 22]. Thirdly, the differentiation of myotubes in these MUs is greatly improved. Finally, generating this system does not need complicated equipment; using MN, Schwann cell, and myoblast cell lines saves a tremendous amount of time and effort (14 days) and greatly improves the reproducibility.

This paper demonstrates both technical and conceptual novelty in assembling the system, and highlights the significance of the bilayer hydrogel which includes the upper Matrigel and the lower micro-molded hydrogel, a key to the successful co-differentiation/maturation of the three cell types that make up a complete and functional NMJ containing topologically aligned and properly innervated myotubes for various biological/physiological interrogations. The features of the generated MU are characterized by the unique morphology and staining with biomarkers, and the biological function/property of MNs, NMJ, and myotubes can be routinely and repeatedly monitored by video recording and calcium images.

## Results

### The concept for designing an architecturally organized and functional 3D MU in vitro model

**Fig. 1:**
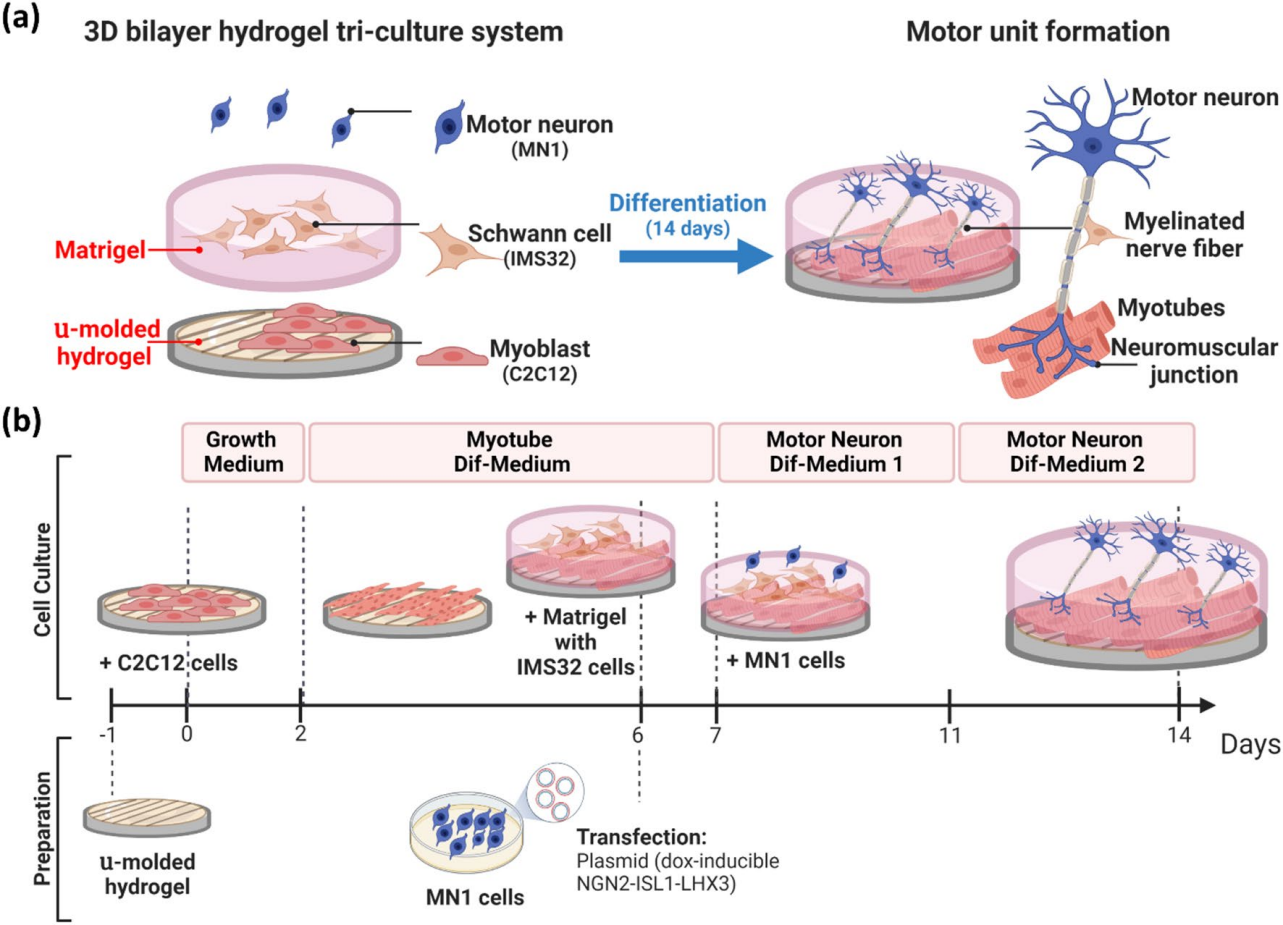
Overview of the 3D bilayer hydrogel tri-culture system for modeling MU. **a** The design of the 3D bilayer hydrogel tri-culture system **b** The procedure for generating the in vitro MU model

**Fig. 2:**
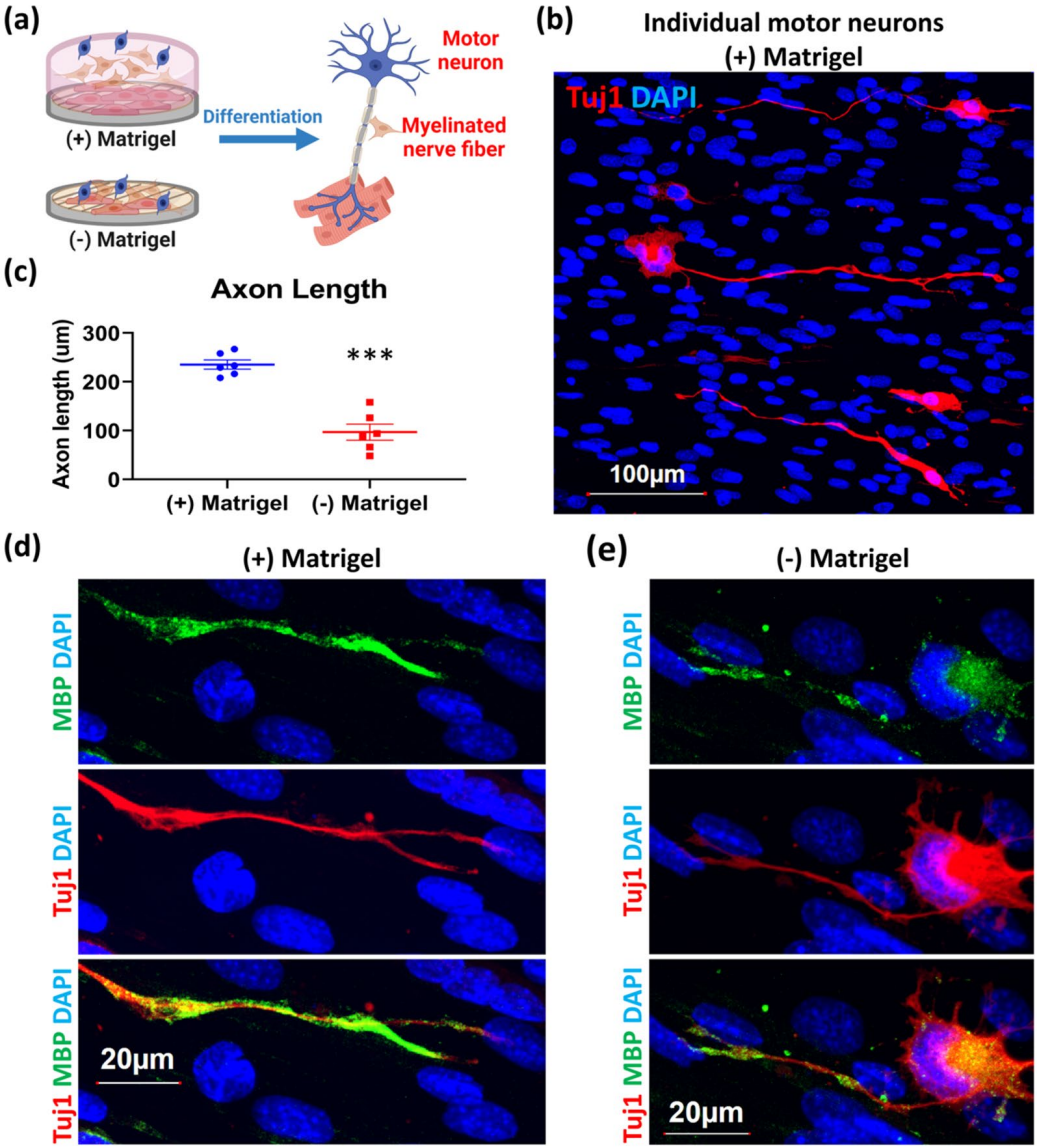
Matrigel-Schwann cell layer contributes to MN differentiation and myelination **a** Experimental scheme for determining the effects of the Matrigel layer. On day 6 of the diagram in Fig. 1b, Schwann cells were mixed with (+) Matrigel group, but not in without (−) Matrigel group. The motor neuron differentiation and myelination were determined on day 14. **b** Immunostaining showed the differentiated motor neurons in (+) Matrigel group. The morphology of motor neurons was observed by neural marker Tuj1 (red), and all the nuclei were detected by DAPI (blue). **c** Quantification data comparing axon length in (+) Matrigel group (n = 6) and without (−) Matrigel group (n = 6). **d** and **e** Immunostaining showing the myelination of an axon and axon terminal with (+) Matrigel (**d**) and without (-) Matrigel (**e**). Myelination was detected using myelin-basic protein (MBP, green). Results were presented as means ± SD, ***p ≤ 0.001, compared with (+) Matrigel group.

**Fig. 3:**
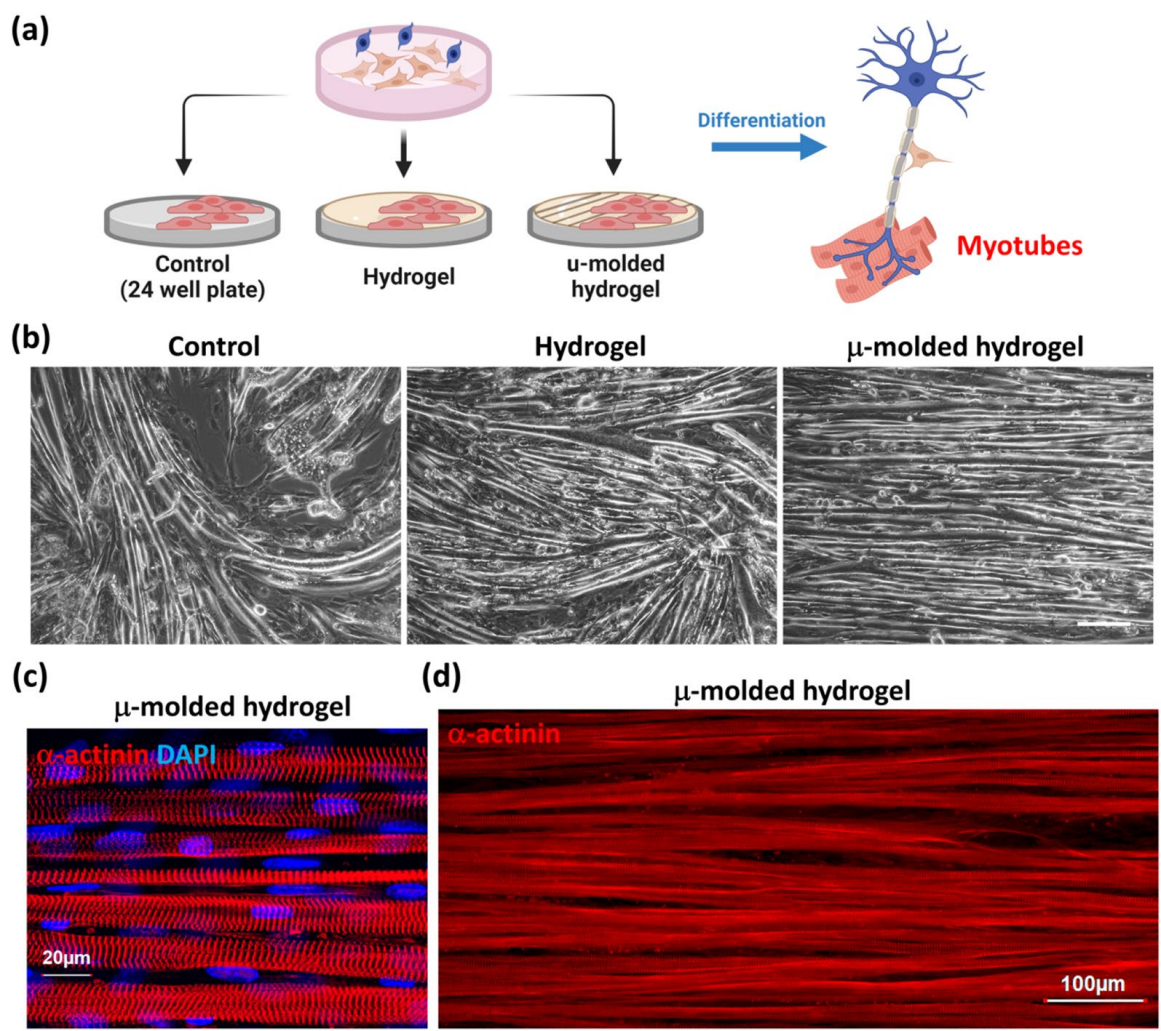
µ-molded hydrogel contributes to myotube differentiation and alignment **a** Experimental scheme for determining the effects of µ-molded hydrogel. On day 0 of the diagram in Fig. 1b, C2C12 cells were seeded on 24 well plate (control), hydrogel, and µ-molded hydrogel. The differentiated myotubes were characterized on day 14. **b** The bright field images showed the morphologies of differentiated myotubes in each group (Scale bar is 200 µm). **c** In µ-molded hydrogel group, immunostaining image characterizing the mature myotubes by the multi-nucleated (DAPI, blue) myocytes with sarcomere structure (α-actinin, red). **d** At lower magnification, Immunostaining of α-actinin (red) showed the aligned, elongated myotubes with a similar width.

**Fig. 4:**
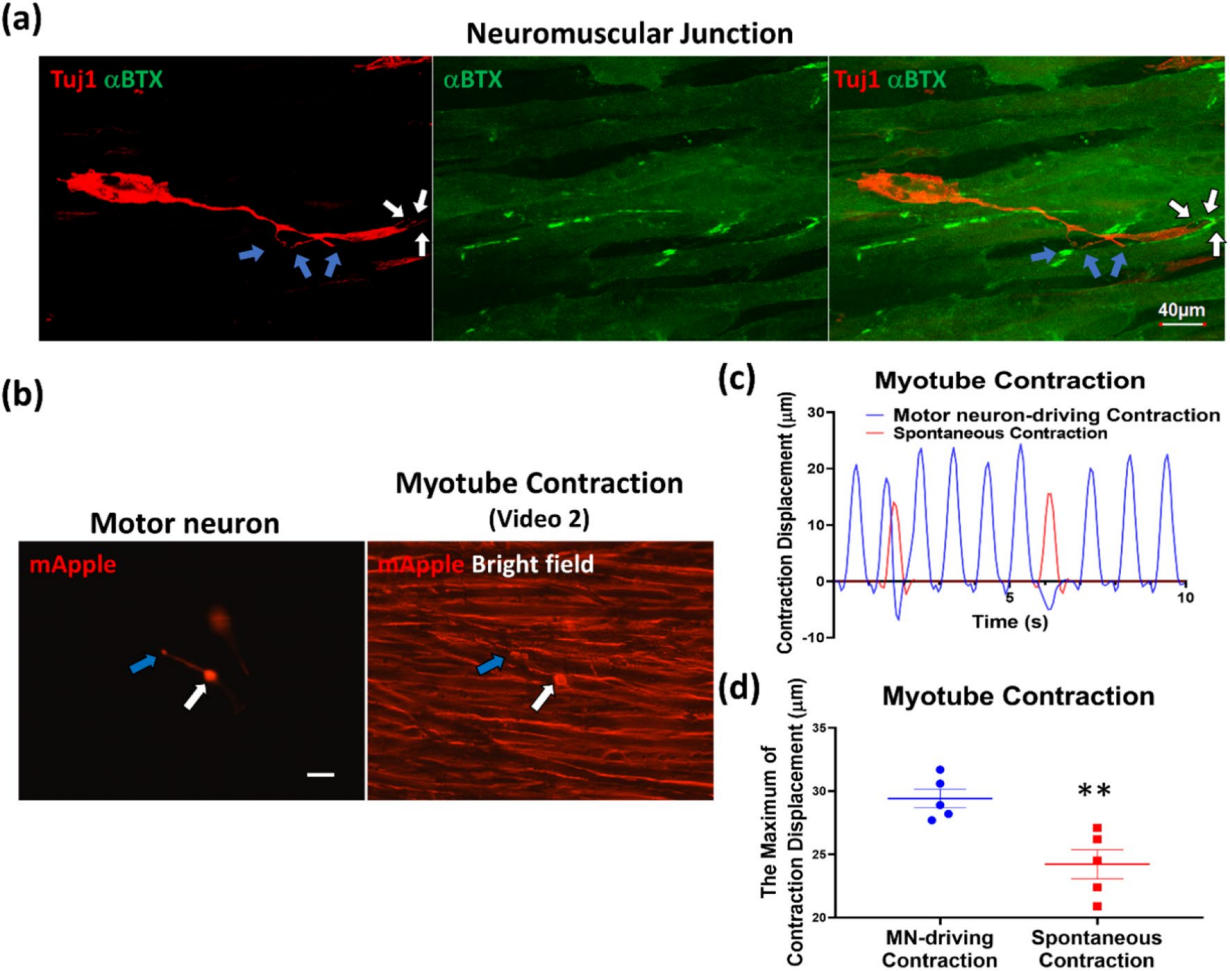
Characterizations of the functional NMJ **a** The immunostaining characterizing the differentiated neuromuscular junction by motor neuron axon terminal (Tuj1, red; white and blue arrows) surrounded with the acetylcholine receptor cluster (αBTX, green signals). **b** The left live-cell image showed the localization of the motor neuron (mApple; white arrow) and its axon terminal (blue arrow) (Scale bar is 200 µm). The right live-cell image from Additional file 3: video S2 showed the MN-driving contraction at the axon terminal (blue arrow) and spontaneous contraction happened without motor neuron. **c** The quantified data showed the amplitude and frequency of the myotube contraction in Additional file 3: video S2. **d** The maximum of myotube contraction displacement was determined in motor neuron (MN)-driving contraction group and spontaneous contraction group (n = 5/group). Results are presented as means ± SD, **p ≤ 0.01, compared with the MN-driving contraction group.

The 3D bilayer tri-culture system is specifically designed for modeling the MUs (Fig. 1a). It is constructed using the three cell types essential to MUs—MN, skeletal muscle and Schwann cells. For the consistency of this system, we have chosen three well studied mouse cell lines for the three cell types, MN1 for MNs, IMS32 for Schwann cells, and C2C12 for myoblasts. It is critical to engineer a microenvironment mimicking the physiological conditions during MU development. This was made possible by the design of a bilayer matrix. (Fig. 2) The custom fabricated µ-molded gelatin hydrogel provides an optimized topological cue on the surface of the bottom layer for myoblast differentiation, orientation and alignment (later Fig. 3). The upper layer Matrigel is designed for several purposes. First, the Matrigel layer physically separates the myoblasts from MN, which enhances axon elongation. Secondly, Matrigel is embedded with Schwann cells to provide an extracellular matrix-like microenvironment for axon myelination (Schwann cell-MN axon interaction) (later Fig. 2d, e). These factors contribute to the formation of NMJs (MN-Muscle cells interaction) (later Fig. 4). After co-differentiation, MN axons readily penetrate the Matrigel layer and reach the myotubes on the µ-molded hydrogel layer to form functional NMJs, while the Schwann cells contribute to the myelinated axons.

The entire procedure is depicted in Fig. 1b. The µ-molded hydrogel was prepared before starting the process. The tri-culture was initiated with seeding C2C12 myoblasts on the µ-molded hydrogel on day 0. After two days of culture in growth medium, myoblasts typically expanded to a > 90% confluency; the culture was then changed to a fresh myotube differentiation medium, followed by media exchange every two days. On day 6, most of the myoblasts began to differentiate. Schwann cells (IMS32) were mixed with Matrigel and added on top of differentiating myoblasts. Also on day 6, in a separate culture dish, MN1 cells were transfected with an expression vector (Plasmid #105842; Addgene) expressing mApple marker and doxycycline-inducible factors Neurogenin 2 (NGN2), ISL LIM Homeobox 1 (ISL1), and LIM Homeobox 3 (LHX3). Growth factors and transcription factors encoded in this plasmid facilitated MN differentiation, resulting in a more uniform MN morphology. Without this plasmid, control MN1-derived neurons varied greatly in morphology as shown in Additional file 1: Supplementary data, Fig S1 [23]. On day 7 > 80% MN1 cells were mApple-positive. These transfected MN1 cells were then plated on top of Matrigel in MN differentiation medium 1 to induce MN differentiation and axon elongation. On day 11, the media was changed to MN differentiation medium 2 which facilitated NMJ formation. MN differentiation medium 2 was changed every two days. By the end of day 14, functional MUs formed and were ready for examination.

### Matrigel layer contributes to MN differentiation and myelination

In this bilayer tri-culture system, the Matrigel layer is a critical element contributing to MN differentiation and myelination. To demonstrate the effect of Matrigel, an experiment was carried out as described in Fig. 2a. On day 6 (described in Fig. 1b), Schwann cells were mixed with (+) or without (−) Matrigel, and MN differentiation and myelination were examined on day 14. As shown in Fig. 2b, the typical multipolar morphology of spinal MN was observed in the group with Matrigel (Tuj1; red). In this group, a single axon was extended from each differentiated MN at one end of the cell body, and multiple dendrites branched out from the other end of the cell body. Importantly, the (+) Matrigel group produced more significant and consistently longer axons as compared to the (−) Matrigel group (Fig. 2c, d, e). Myelination of axons by Schwann cells was examined by immunostaining with an antibody against myelin basic protein (MBP; green). As expected, MBP signals were detected only on the axons and axon terminals but not on the cell body in the (+) Matrigel group (Fig. 2d). In contrast, in the (−) Matrigel group, most of MBP signals were detected around the cell body with partial coverage of axons and axon terminals (Fig. 2e). Taken together, these data demonstrate that the Matrigel layer, containing Schwann cells, is critical for efficient MN differentiation, axon elongation, and myelination.

### µ-molded hydrogel contributes to myotube differentiation and proper alignment

The µ-molded hydrogel is critical for myoblast differentiation and alignment [24, 25]. To demonstrate the effects of µ-molded hydrogel, C2C12 cells were seeded into an untreated 24 well plate (uncoated Control), a hydrogel-coated plate, or a µ-molded hydrogel coated plate as described in Fig. 3a. The system was maintained as described in Fig. 1b, and differentiation of myotubes was examined on day 14. Myoblasts proliferate and differentiate into myocytes, which then fuse into multinucleated myotubes and express skeletal muscle markers such as sarcomeric α-actinin. α-actinin specifically marks Z-disks, a (Fig. 2) myotube feature that defines individual functional units of skeletal muscle known as sarcomeres. As shown in Fig. 3b, myotube formation was observed in all the experimental groups, but myotube morphology differed across these conditions. In the control group and the hydrogel group (left and middle panels; Fig. 3b), myotubes appeared to lack proper alignment. Further, most of the myotubes in the control group were detached from the surface on day 12 when contraction is typically observed. In contrast, the µ-molded hydrogel group showed well differentiated and aligned myotubes (right panel, Fig. 3b) that exhibited expected contractibility (contractility recording in Additional file 2: Video S1).

To further evaluate the organizational features of mature myotubes in the µ-molded hydrogel group, we labeled the 3D culture with an antibody against α-actinin (Fig. 3c, d). The mature myotubes in the completed tri-culture resemble in vivo skeletal muscle fibers. As shown in Fig. 3c, the α-actinin signals were concentrated at the Z-disks which cross-linked the thin filaments in adjacent sarcomeres, the basic contractile units of myotubes. Further, in contrast to the typical central localization of nuclei in immature myotubes, the nuclei in these mature myotubes (DAPI; blue) were flattened and adjacent to the surface of the myotubes, as expected in mature cylindrical structure of myotubes. Finally, under a lower magnification (Fig. 3d), these striated and aligned myotubes had a similar width, approximately 15–25 µm, and were well elongated. Taken together, the µ-molded hydrogel contributes to the alignment of myotubes. The overall design of this 3D bilayer hydrogel tri-culture system facilitates the formation of skeletal muscle with cylindrical, aligned, striated, unbranched and extremely long myotubes (more than 700 µm).

### Characterization of functional NMJs in the 3D culture system

In addition to characterizing the morphological features and biomarkers of the 3D culture, we also evaluated the physiological function of this MU system. NMJs were studied using a neural marker Tuj1 (Red) to label the pre-synaptic MNs, and an AChR marker αBTX (green) to label the post-synaptic compartments. As shown in Fig. 4a, two groups of small branches of the axon terminals form specialized pre-synapses (White and blue arrows, left panel), suggesting that the MN can innervate two myotubes (lower and upper). Importantly, while approximately 8–9 myotubes can be seen in this field, only 2 of these myotubes, which are located adjacent to axon terminals, exhibit clear AChR clusters (stained with αBTX, Middle panel). These AChR clusters are detected near the MN in this filed, especially at its axon terminal (Right panel). This provides the evidence that this MN recruits AChRs to its axon terminal to form NMJ. Moreover, NMJ formation is progressively detected, from differentiation day 12 (Additional file 1. Supplementary data, Fig S2) to day 14 (Fig. 4a), including enlarged axon terminals, small branches, and recruited AChR clusters. Together, these results show that this 3D culture system provides an in vitro model for studying NMJ development and formation.

To further examine the functionality of NMJs in this tri-culture system, we performed two functional assays: contractility, and calcium imaging. The following section describes contractility of functional MUs. Additional file 2: Video S1 recorded myotube contraction under bright field illumination. The location of three MNs (determined based on mApple signal) in this field are indicated with white arrows as shown in Additional file 1: Supplementary data, Fig. S2, while five contracting myotubes are indicated with blue arrows. The myotubes contracted in response to MN stimulation, indicating the formation of functional NMJs. Notably, the lower MN drove two fast-contracting myotubes, while the upper two closely localized MNs drove synchronized slow-contracting myotubes. This indicates the formation of individual MUs and functional NMJs that mediate MN-driven myotube contraction.

To further illustrate MN-driven myotube contraction, we seeded mApple-marked MNs in an ultra-low density (2 X 10^2^ cells/cm^2^) on day 7. Additional file 3: Video S2 recorded the contractility in this experiment. A typical image was taken, shown in Fig. 4b. The left panel of Fig. 4b shows the soma (white arrow) and enlarged axon terminal (blue arrow) of a MN. The right panel of Fig. 4b was extracted from Additional file 3: Video S2 which recorded the contracting myotube and the MN under bright field illumination using a fluorescence filter for Texas Red to simultaneously view mApple-marked MNs and myotubes in the same field. The contraction displacement in Additional file 3: Video S2 was quantified as shown in Fig. 4c. Myotube contraction occurred with the maximum displacement localized at the axon terminal (Additional file 3: Video S2 and Fig. 4c); further, the contraction frequency of the MN-driving myotubes was significantly greater than that of spontaneous contraction (Additional file 3: Video S2 and Fig. 4c, d). This provides further evidence that MNs drive myotube contraction in this 3D tri-culture system, indicating the formation of functional NMJs in MUs.

### Ca2 + imaging of MNs and myotubes

We next demonstrated the physiological function of two key components of the MUs, MNs and myotubes, using a conventional calcium imaging method to probe the functionality of neurons (including MNs) and muscles (myotubes in MUs). Physiologically, MNs and myotubes respond to stimuli that evoke neuronal firing and muscle contraction [20] both with increases in the intracellular calcium concentration ([Ca2 +]i). Therefore, calcium imaging provides an appropriate tool to study the functions of MNs and myotubes. We employed a fluorescent, ratiometric calcium indicator, fura-2AM, to detect changes in [Ca2 +]i following exposure to a depolarizing stimulus (for MNs) or neurotransmitter acetylcholine (ACh) (for myotubes). Changes in [Ca2 +]i are represented by changes in the 340nm/380 nm ratio. It appeared that depolarization with 50 mM extracellular K + or treatment with 10 µM ACh evoked measurable changes in [Ca2 +]i in MNs and myotubes, respectively. As expected, the Schwann cells did not show any changes in [Ca2 +]i under these treatments, indicating that the added Schwann cells in the tri-culture did not exhibit inappropriate response. This further supports the specificity of this improved tri-culture model with respect to physiologically relevance and the specific functional response of each component.

**Fig. 5:**
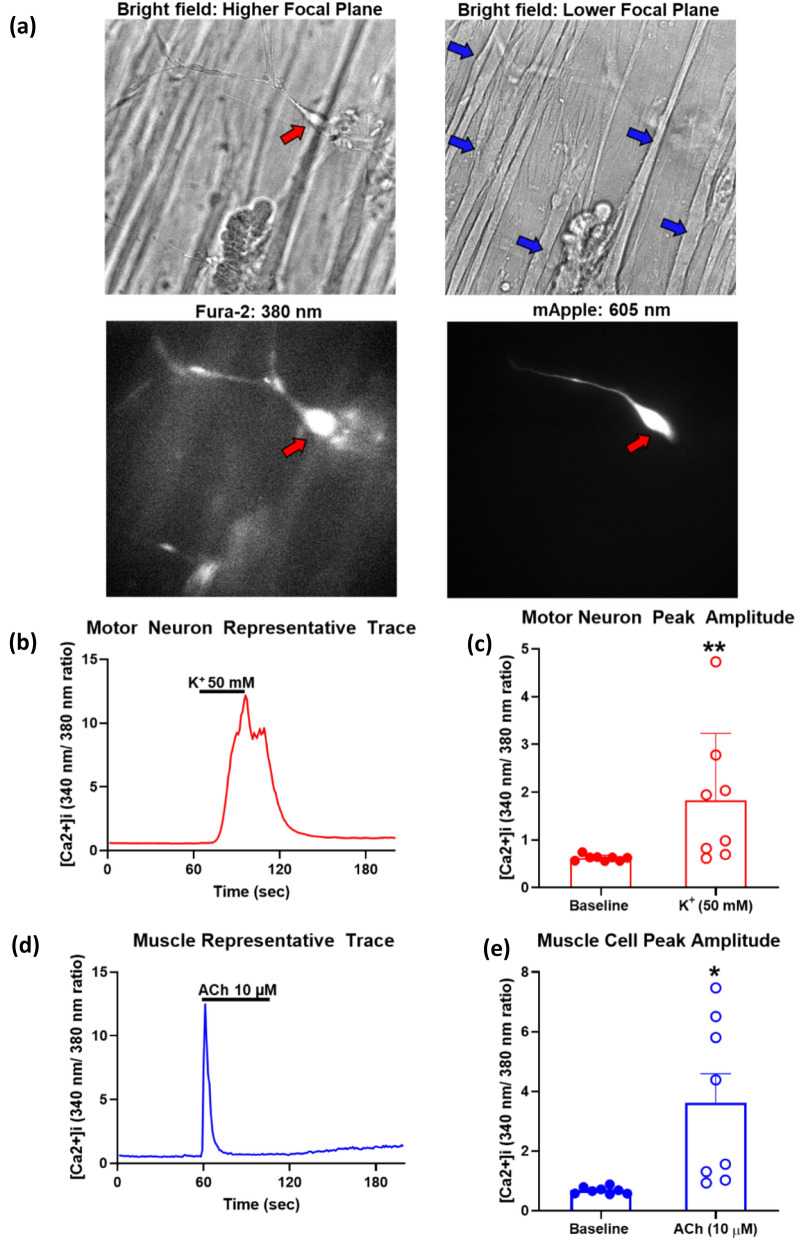
Ca^2+^ imaging **a** Representative field of a tri-culture in bright field illumination (top left and right), Fura-2 fluorescence (380 nm; bottom left), and mApple fluorescence (605 nm; bottom right). Red arrows indicate a motor neuron and blue arrows indicate myotubes. **b** Representative trace shows a typical response observed in MNs during stimulation with 50 mM K + . **c** Bar graph displays MN baseline and depolarization-evoked 50 mM K +) peak amplitude [Ca2 +]i (n = 8). *P* = 0.008. Wilcoxon matched-pairs signed rank test. **d** Representative trace shows a typical myofiber response to ACh (10 μM) stimulation. **e** Bar graph displays myotube baseline and ACh (10 μM) evoked peak amplitude [Ca2 +]i (n = 8), * *p* = 0.02 paired Student’s t-test. Data presented are means ± SD and * *p* < 0.05, ** p < 0.01.

Figure 5a depicts representative images of a single field showing bright field illumination (top left and right), fura-2 fluorescence (bottom left), and mApple fluorescence (bottom right). Bright field images show that MNs (red arrows) rest on the top Matrigel layer (top left) and myotubes (blue arrows) on the bottom hydrogel layer (top right) of the 3D tri-culture, mimicking the architecture of MUs. Fura-2AM is taken up by, and labels MNs, Schwann cells, and myotubes. MNs are identified by their expression of mApple, as described earlier. Figure 5b shows a representative trace of a MN response to a 30 s, 50 mM K^+^ stimulation. A high extracellular K + concentration depolarizes neurons by opening voltage-gated Ca2 + channels, resulting in Ca2 + influx [21]. A MN response appears to have a peak in signal followed by several oscillations at peak amplitude and then decrease to baseline. Across independent experiments (n = 8 fields), 70% MNs responded (43/62 cells) to 50 mM K + stimulation. The depolarization-evoked response rose from a basal 340/380 ratio of 0.62 ± 0.06 to peak of 1.83 ± 1.41 (Fig. 5c).

Figure 5d shows a representative trace of a typical myotube response stimulated with 10 µM Ach for 60 s. At this concentration, ACh has been shown to induce intracellular Ca2 + release and contraction [22, 23]. Myotubes responses exhibited a sharp increase in [Ca2 +]i immediately upon stimulation and then a rapid decline back to the baseline. Across independent experiments (n = 8 fields), 89% of myotubes responded (24/27 cells) to ACh stimulation. The ACh-evoked response rose from a basal 340/380 ratio of 0.68 ± 0.12 to peak of 3.62 ± 2.73 (Fig. 5E). Furthermore, in at least two independent experiments myotubes also exhibited physical contraction when stimulated with ACh (Additional file 4: Videos S3 and Additional file 5 Videos S4). Spontaneous contraction was also observed in some tri-cultures (Additional file 4: Video S3). These data indicate that the key functional components of a MU, MNs and myotubes, exhibit measurable and significant increases in [Ca2 +]i in response to physiological stimuli.

## Discussion

This 3D bilayer tri-culture system is specifically designed for modeling MUs that can be used to study a wide spectrum of biological problems associated with NMD and in pharmacological studies such as drug screening. These experiments demonstrate the benefits of the bilayer hydrogel design. This design exploits an upper layer, Matrigel layer, that contributes to MN differentiation and timely myelination, and a lower layer, µ-molded hydrogel, that contributes to myotube differentiation, alignment and innervation. The feature of this MU model is demonstrated according to multiple criteria including morphological characteristics, essential biomarkers of NMJ, and MU function such as contractility and calcium signaling.

There are several positive features of this new model. First, this model contains the three cell types of a MU, including MNs, Schwann cells, and myotubes, and all three cell types are well differentiated and functional. Secondly, the bilayer µ-molded hydrogel/Matrigel provides a critically needed micro-environment mimicking the physiological condition of a developing MU. Specifically, MNs and muscle cells are separated initially, and then make contact for co-differentiation and synapse formation only when MN axons are myelinated by Schwann cells and penetrate to the muscle layer where myotubes are maturing. At this stage, MN axons innervate myotubes to form NMJs. For this to happen, Matrigel provides an environment resembling the extracellular matrix that separates MNs from muscle cells. This layer embeds Schwann cells for axon myelination when MN axons extend through this layer. Further, µ-molded hydrogel forms a surface area facilitating differentiation and the alignment of myotubes, which is critical to the formation of organized myotubes. A third advantage of this 3D system is that multiple parameters of individual MUs can be examined and quantified, such as axon length and the frequency of myotube contraction. Moreover, axon myelination, mature myotube, NMJ formation can all be assessed by immunostaining. These could be useful in studying pathological conditions associated with various NMDs, such as axonal regression, demyelination, cell death, muscle atrophy, and degradation. Importantly, Ca^2+^ imaging can be used to monitor the biological function/property of individual MNs and myotubes. Finally, by manipulating MNs or myoblasts, it is possible to study fundamental questions related to MUs and to model NMDs in vitro*.* In our previous study [26], we employed a simpler (without Schwann cells and Matrigel) in vitro MU model containing only MNs and myotubes on µ-molded hydrogel to study NMJ formation. Using this earlier system, we have successfully demonstrated that the expression level of a retinoic acid signaling protein, Cellular Retinoic Acid Binding Protein 1 (CRABP1) in MNs affected the length of axons and AChR cluster formation. The requirement for CRABP1 in MU functionality in vivo was validated in *Crabp1* knockout mice and confirmed using gene rescue experiments. Therefore, this 3D MU system can be a powerful tool to efficiently identify molecular players and dissect signaling pathways critical to formation and function of MUs or NMJs. For a practical consideration (time and effort), this system provides a reliable and efficient MU model for numerous studies including physiological and pharmacological investigations.

The formation of functional NMJs in the 3D tri-culture system is also supported by data shown in our previous study [26] where a similar, but simpler (only two cell types, MN and C2C1_2_), version of co-culture system was reported. This earlier study has detected the expression of pre-synaptic vesicle protein SV2 and post-synaptic AChR in NMJs [26]. The expression of synaptic vesicle glycoprotein-2 would indicate the ability of MNs to release neurotransmitter from the axon terminals. To improve the physiological relevance of this earlier model, in this current study, we added the Matrigel layer embedded with a third cell type important for NMJ, i.e. Schwann cells. This has apparently improved this model as demonstrated in multiple measurements including extended axon length, myelinated axons, and axon terminals. In the NMJ of this tri-culture model (Fig. 4a), the presence of two groups of small branches of the axon terminal (White and blue arrows, left panel) would suggest that the MN can innervate multiple myotubes. The morphology of small branches would indicate the potential of NMJ to form a pretzel-shaped structure, a classic morphological feature of NMJs as shown in murine models (Fig. 4a, left panel). In the post-synaptic compartment, the expression of AChR is clearly detected, predominantly near the MN especially at its axon terminal (Fig. 4a, Additional file 1: Supplementary data, Fig S2), indicating that the MN recruits AChRs to its axon terminal to form AChR clusters. The functionality of NMJ is demonstrated using a conventional live imaging method that detects the dynamics of Ca2 + reflecting the functional response of neurons (MN in this case) and muscles (myotubes in this case). Ca2 + imaging data validate the relevant responses of key components in the MUs—increases in intracellular Ca2 + upon high potassium (KCl) or acetylcholine stimulation for MNs (electrophysiological response), or myotubes (contractility), respectively, but not in Schwann cells as predicted. Therefore, each cellular component (MNs, myotubes, and Schwan cells) appropriately exhibits its biological response (or lack thereof) to stimuli, supporting the physiological relevance of this improved, tri-culture model. As shown in Fig. 4b and Additional file 3: video S2, both myotube contraction and the maximum displacement occurred specifically at the axon terminal. Additionally, the contraction frequency of the MN-driven myotubes was fivefold greater than that of the spontaneous contraction of myotubes without MNs nearby (Fig. 4c and Additional file 3: video S2). Taken together, these results support the formation of functional NMJs in the 3D tri-culture.

We recognize that there remain limitations in the application of this in vitro 3D culture MU model. The purpose of using cell lines to generate this MU model is for the sake of consistency, ease in culture procedure and timesaving. It remains to be determined whether appropriate human cell lines, or stem cell systems, such as iPSC differentiated MNs or muscle cells, can be similarly optimized for this system [14]. Secondly, in Ca^2+^ imaging experiments, due to the thickness of Matrigel layer, it will be challenging to collect clear signals from both MNs and myotubes at the same time because the cells reside in different layers (Fig. 5a). This would limit real-time assessment of MN excitation together with MN-driven myotube contraction. However, advanced imaging tools may be useful to improve temporal resolution of the assay. While the percentage of MNs and myofibers that responded to physiological stimuli with an increase in [Ca^2+^] was quite high, the amplitude of the responses was variable. Possible explanations for this variability likely result from the challenges of accurately correcting background fluorescence in a 3D population of cells and variable drug application and dye loading through the layers of the gel matrix. These issues might be addressed in future experiments by using genetically encoded Ca^2+^ indicators targeted to specific cell types and local drug application by micropipette. Overall, the Ca^2+^ recordings described here demonstrate the functional viability of this new model system.

It is worthy to note the well differentiated and organized mature myotubes in this 3D bilayer hydrogel tri-culture system. The mature myotubes are similar to skeletal muscle fibers [27], including their morphology, similar width, length and a cylindrical, aligned, striated, and unbranched shape. The µ-molded hydrogel contributes to the alignment of myotubes and the Matrigel may provide the extracellular matrix-like microenvironment to improve myotube maturation. Additionally, the Matrigel also protects the contracting myotubes, preventing them from detachment in a long-tern culture.

## Conclusion

We report a procedure to engineer a structurally organized in vitro 3D MU model that can be reliably generated within a reasonably short period of time. This MU model is validated with regards to morphology, essential biomarkers and the function of a MU including contractility and calcium signaling. Three key advantages are noted in this new model. First, the entire MU (single MN) can be examined, and subcellular/functional details can be studied. Secondly, the included Schwann cells allows proper myelination to occur, enabling detailed studies of MN regulation with regards to NMJ development/maturation and maintenance. Thirdly, using this system, it is possible to generate various in vitro disease models by manipulating each relevant cell type. Thus, this new system is appropriate not only for basic research, but also for disease modeling and drug screening.

## Methods and materials

### Cell lines and its culture condition

The mouse C2C12 myoblast cell line was purchase from ATCC (Cat. no. CRL-1772). The mouse motor neuron cell line MN1 were kindly provided by Ahmet Hoke (The Johns Hopkins University, Baltimore, MD). The mouse Schwann cell line IMS32 were purchased from CosmoBio Co., LTD (Cat no. PMC-SWN-IMS32C). The three Cell lines were maintained in high glucose Dulbecco’s Modified Eagle Medium (DMEM) with 2 mM L-glutamine (Thermo Fisher Scientific, cat. no. 11965–092) supplemented with 10% FBS (ALTANTA Biologics, cat. no. S11150), 100 U/mL penicillin and 100 mg/mL streptomycin (Thermo Fisher Scientific, cat. no. 15140–122). Cells were maintained at 37 °C in a humidified 5% CO_2_ cell culture incubator. The C2C1_2_ Cells were sub-cultured using 0.25% trypsin (Thermo Fisher Scientific, cat. no. 25200–056) upon reaching approximately 70% confluence. The MN1 cells were sub-cultured using 0.05% trypsin (Thermo Fisher Scientific, cat. no. 25200–054) upon reaching approximately 90% confluence. The IMS32 cells were sub-cultured using 5 mM EDTA (Thermo Fisher Scientific, cat. no. 15575–020) upon reaching approximately 70–80% confluence.

### The procedure for the 3D bilayer hydrogel tri-culture system is described in Fig. 1b

#### Stamp fabrication

PDMS stamps were fabricated as described elsewhere [24, 26, 28]. Briefly, the silicon wafer mold was treated with trichloromethyl silane (Millipore Sigma, cat. no. 75-79-6) vapor for 30 min as a releasing agent. PDMS mixture (PDMS: curing agent = 10:1, Sylgard 184, Dow Corning) was poured onto the master mold and cured for 2 h. at 60 °C. The cured PDMS was excised from the master mold and trimmed with a clean razor blade to generate the stamp. The PDMS stamp was then cleaned with oxygen plasma (irradiation intensity: 200 W) for 10 min.

#### Micro-molded (µ-molded) hydrogel fabrication

Under sterile conditions, prepare a 10% solution of gelatin A (Sigma, cat. no. G1890-100 g) was prepared in sterile ddH_2_O. The solution was placed on a rotating rack at 65 °C for 10 min or until thoroughly dissolved, then removed from rotation to allow bubbles to rise out of the solution for 10 min. A 65 °C hotplate (Apollo; Digital Heating and Chilling plate) was used to create the µ-molded hydrogel and adhere it to the cover glass (Fisher Scientific, cat. no. 1254580), so µ-mold PDMS stamps, gelatin, and coverslip glass were warmed to 65 °C before molding. 18 µl of gelatin solution was carefully pipetted directly onto the stamp, then quickly place the cover glass on top of the gelatin droplet ensuring full coverage of the glass. The stamps were remove from the hotplate and the entire sandwich was allowed to cool at room temperature for 30 min. Under sterile conditions, the cover glass and hydrogel was carefully peeled off from the stamp using forceps and placed in a 24 well plate. The hydrogels were exposed to UV for 5 min.

The gelatin hydrogel was crosslinked with a 5% transglutaminase solution for 18 h. 1 g of powdered transglutaminase was mixed with 19 ml of autoclaved ddH_2_O under sterile conditions. The enzyme was placed on a rotating rack at 50 °C for 10 min to dissolve and allowed to cool at room temperature before filtration. The transglutaminase solution was filtered through a 0.2 µm filter. After hydrogels have had 5 min under UV, they were ready for the addition of transglutaminase. Leave the 24 well plate covered in the hood at room temperature for 18 h. After 18 h, transglutaminase was suctioned out. The hydrogel was then rinsed three times by adding autoclaved ddH_2_O to the well. On the last rinse, the 24 well was placed under UV uncovered for 5 min. Following UV exposure, the 24 well plate can be covered, placed in a sterile bag, and stored at 4 °C until use.

#### MN1 transfection

Plasmid CLYBL-(Ef1a-SBP-LNGFR-T2A-mApple)-(CAG-rtTA)-(TRE-hNIL)) was purchased from Addgene (cat. no. 05842). MN1 cells transfection was performed by Lipofectamine 3000 (Thermo Fisher Scientific, cat. no. L3000-015) following the manufacturer’s instruction. MN1 cells were added to the tri-culture system 1 dayafter transfection.

#### Cell culture in the system

On day 0, 2 × 10^4^ C2C12 cells/cm^2^ were seeded on hydrogels in 24-well plates and cultured in growth medium. Once confluent (about 2 day), myotube differentiation medium (DMEM with 2% horse serum (Thermo Fisher Scientific, cat. no. 5H30074.2), 100 U/mL penicillin, 100 mg/mL streptomycin and 2 mM L-glutamine) was added. C2C12 were differentiated for 4 days and the myotube differentiation medium was replenished every 2 days.

On Day 6, Schwann cell line IMS32 was trypsinized by 0.05% trypsin and re-suspended in cold myotube differentiation medium in the density of 4 × 10^4^ cells/ml. Schwann cells were mixed with Matrigel in a 1-to-1 ratio and then kept on ice. Matrigel Matrix (Corning, cat. no. 356255) was thaw in ice in cold room over night before use. The medium in the system was removed and 18 µl of the mixture was added on the center of the hydrogel. The system was kept in 37 °C incubator for 3 min, and added the fresh myotube differentiation medium.

On day 7, the transfected 1 × 10^3^ MN1 cells/cm^2^ were added to each well of 24-well, on top of the differentiated C2C12 cells. MN1 cells were previously transfected with either empty vector control or GFP-Crabp1 prior to seeding onto C2C12 cells. The co-culture was maintained in MN differentiation medium 1 (DMEM media with 100 U/mL penicillin, 100 mg/mL streptomycin, 2 mM L-glutamine, 1% MEM non-essential amino acids (NEAA; Thermo Fisher Scientific, cat. no. 11140–050), 2 µg/ml doxycycline (Sigma, cat. no. D9891) and 10 um Y27632 Dihydrochloride (R&D, cat no. 1254/1) for 4 days, then changed to MN differentiation medium 2 (MN differentiation medium 1 without Y27632 Dihydrochloride). The MN differentiation mediums was replenished every two days.

#### Immunostaining

For Immunostaining, the tri-culture slides were fixed in 100% methanol for 5 min at – 20 °C. After three times of 5 min 1XPBS wash, 2% BSA was added for 1 h at room temperature for blocking. Primary antibodies, α-actinin (1:100; Cell Signaling, cat. no. 6487S), Tuj1 (1:1000; R and D, cat. no. MAB 1196) or myelin basic protein MBP (1:500; R and D, Cat. No. MAB42282) in 1XPBS were incubated overnight at 4 °C. After three times of 5 min 1XPBS wash, fluorescent secondary antibodies (1:1000), and µBTX conjugated to a fluorescent dye (1:400; Biotium, Cat No. 00005) were incubated for 2 h in dark at room temperature. The DAPI (1:1000; Thermal Fisher Scientific, Cat. No. 62248) in 1 × PBS were incubated for 10 min in dark at room temperature. The slide then was washed three times of 5 min 1xPBS. Z-stack images were captured on Olympus Fluoview FV1000 BX2 Upright Confocal.

#### Calcium imaging

Tri-cultures on 12.5 mm glass coverslips were briefly washed in HEPES Hanks’ Buffer Solution (HHBS) (5.6 mM glucose, 20 mM Hepes, 3 mM NaHCO_3_, 0.3 mM Na_2_HPO_4_, 0.4 mM KH_2_PO_4_, 137 mM NaCl, 5.0 mM KCl, 4.9 mM MgCl_2_, 4.1 mM MgSO_4_, 1.26 mM CaCl_2_, pH = 7.4) and then the coverslip was incubated with fura-2 AM in 0.04% pluronic acid dissolved in HHBS to a final concentration of 20 μM for 30 min at 37 °C. After loading indicator, coverslips were washed in fresh HBSS for 10 min at 37 °C and then transferred to an imaging chamber for microscopy experiments.

Fura-2 imaging was performed on an Olympus IX71 microscope using a 20X objective lens. Excitation wavelength was selected with a galvanometer-driven monochromator (8 nm slit width) coupled to a 75 W xenon arc lamp (Optoscan; Cairn Research). Intracellular Ca2 + concentration ([Ca2 +]i) was monitored by sequential excitation (1 Hz) of fura-2 at 340 and 380 nm. A single image was acquired at 610 nm to identify mApple expressing MNs in the field. Fluorescent images were captured using a cooled charge-coupled device camera (Cascade 512B; Roper Scientific) and Metafluor acquisition software (Molecular Devices). All fura-2 experiments were performed in HBSS buffer at room temperature. To depolarize MNs the extracellular K + concentration was increased to 50 mM with K + exchanged reciprocally for Na + in HHBS. For myotube stimulation 10 μM ACh was applied by bath perfusion. A 60 s baseline in HHBS was acquired before stimulation of each coverslip. High K + was applied for 30 s and ACh for 1 min. After stimulus application, a washout of 60 s was performed. Background images were acquired after each experiment; the coverslip was wiped clean, then 20 frames were acquired using the same parameters as above.

### Calcium imaging data analysis

Images were processed using MetaFluor Analyst to draw regions of interest (ROIs), subtract background, and generate 340/380 ratios. MN ROIs were selected based on mApple expression and further verified by morphology by a blinded expert. Differentiated MNs each has a cell body and a single enlarged axon terminal. Myotube ROIs were selected based on morphological criteria; myotubes exhibit a distinct elongated, skeletal muscle fiber-like morphology. Given that myotubes can span the total length of the field, three ROIs for each individual myotube were drawn and averaged to represent a single cell.

Peak amplitude analysis was performed by comparing the average baseline [Ca^2+^]_i_ to the maximum reached after stimulation. Only responding cells were included in peak amplitude analysis. A response was defined by the following criteria: (1) a signal intensity of at least 450 (16-bit scale) after background subtraction, and (2) a peak [Ca^2+^]_i_ at least two standard deviations (SD) above baseline (60 s average). Criterion “1” was empirically determined to exclude cells with poor signal to noise ratio. [Ca2^+^]_i_ calculations were performed in Microsoft Excel by averaging 340/380 intensity ratios for MNs or myotubes in the field that responded to stimulation.

### Statistical analysis

Data were analyzed using the Student’s t test. Statistical analyses were performed using Prism 8.0 (GraphPad, CA). All tests were performed at a significance level of *p* ≤ 0.05, and data are presented as mean ± standard error or mean ± SD as indicated. For statistical analyses of calcium image, MN and myotube data were assessed for normality using the D'Agostino-Pearson normality test. Myotube data exhibited normality, whereas MN did not. Therefore, myotube data was subjected to paired Student’s t-test and MN data were subjected to the non-parametric Wilcoxon signed-rank test^8^. For each experiment “n” was defined by a single microscopic field on a single coverslip. Percent responders was calculated using number of responders/total MNs or myotubes in the field.

### Supplementary Information

**Additional file 1: Supplementary data. Fig. S1.** The morphology of less differentiated MN1-derived MNs. **Fig. S2.** Image of 3D tri-culture on differentiation day 12. **Fig S3.** Relative positions of MNs and contracting myotubes for video 1.

**Additional file 2: Video S1. **Shows the MNs driving-myotube contraction under a bright field.

**Additional file 3: Video S2.** Shows an individual MU and the MN driving-myotube contraction under a bright field using a fluorescence filter for Texas Red.

**Additional file 4: Video S3.** Tri-culture with spontaneous and ACh induced contraction. Images show the 340 nm channel (left) and pseudo-color of the fura-2 340/380 fluorescence ratio (right). Increases in [Ca2+]i are indicated by red in the pseudo-color image. Initially, spontaneous contractions are visible at baseline. ACh (10 µM) application stimulated physical contraction (left) and an increase in [Ca2+]i.

**Additional file 5: Video S4.** Tri-culture with ACh induced contraction. Images show the 340 nm fluorescence (left) and pseudo-color of the fura-2 340/380 intensity ratio (right). Increases in [Ca2+]i are indicated by red in the pseudo-color image. ACh (10 µM) evoked physical contraction (left) and an increase in [Ca2+]i.

## Data Availability

All data are available upon request.

## Abbreviations

MU: Motor unit
MN: Motor neuron
NMD: Motor neuron disorder
3D: Three-dimensional
NMJ: Neuromuscular junction
Ach: Acetylcholine
ESC: Embryonic stem cell
Ipsc: Induced pluripotent stem cell
µ-molded: Micro-molded
NGN2: Neurogenin 2
ISL1: ISL LIM Homeobox 1
LHX3: LIM Homeobox 3
MBP: Myelin basic protein
AChR: Acetylcholine receptor
