# Supplementary material for: A novel 3D bilayer hydrogel tri-culture system for studying functional motor units
Source: Cell Biosci. 2023 Sep 12;13:168. doi: 10.1186/s13578-023-01115-2 (PMC10496371; doi:10.1186/s13578-023-01115-2)
Supplement: Supplementary file 1 — Additional file 1: Supplementary data. Fig. S1. The morphology of less differentiated MN1-derived MNs. Fig. S2. Image of 3D tri-culture on differentiation day 12. Fig S3. Relative positions of MNs and contracting myotubes for video 1. [file 13578_2023_1115_MOESM1_ESM.docx]

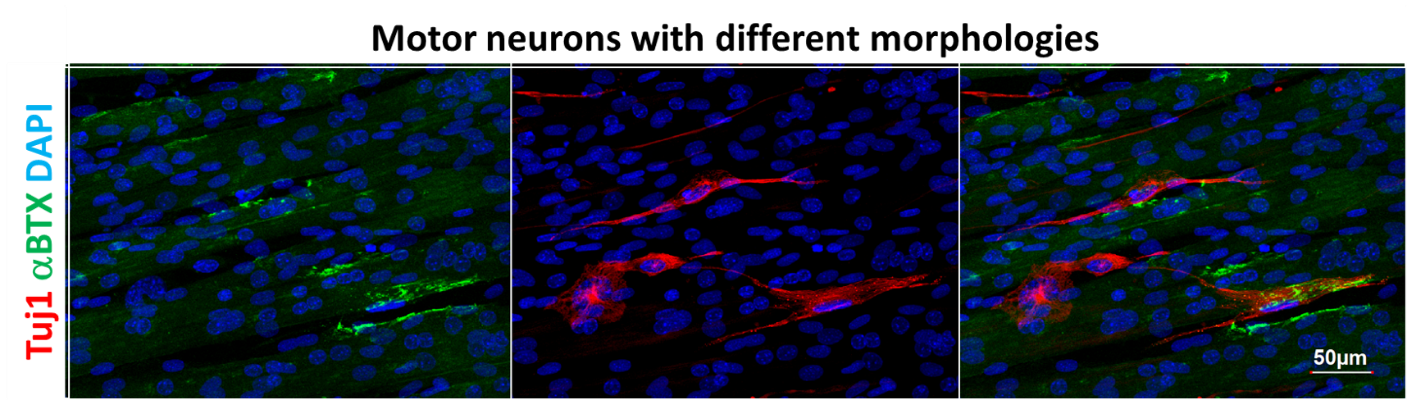
**Additional file Figures**

**Additional file 1: Fig 1. The morphology of less differentiated MN1-derived MNs.**

MN1 cells that were differentiated without transfection of the plasmid CLYBL-(Ef1a-SBP-LNGFR-T2A-mApple)-(CAG-rtTA)-(TRE-hNIL) as a control comparison. Expression of this plasmid results in consistent and fuller differentiation of MN1 cells. MN differentiation was monitored on day 14. Immunostaining showed less differentiated morphology of these control MN1-differentiated cells, as indicated by the neural marker Tuj1 (red) and the expression of AChR on myotubes by αBTX (green).


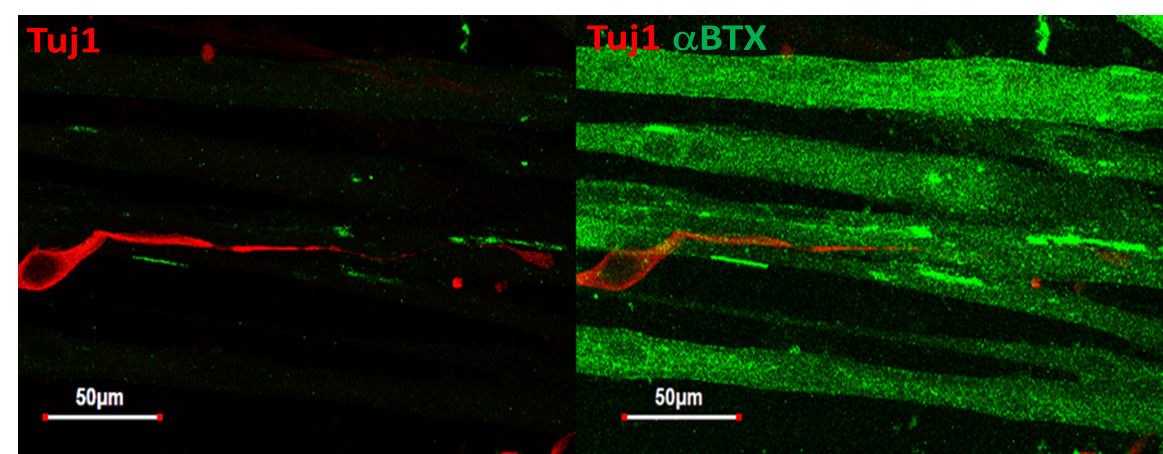


**Additional file 1: Fig 2. Image of 3D tri-culture on differentiation day 12.**

Immunostaining image to monitor neuronal marker Tuj1 (red) and AChR marker αBTX (green).


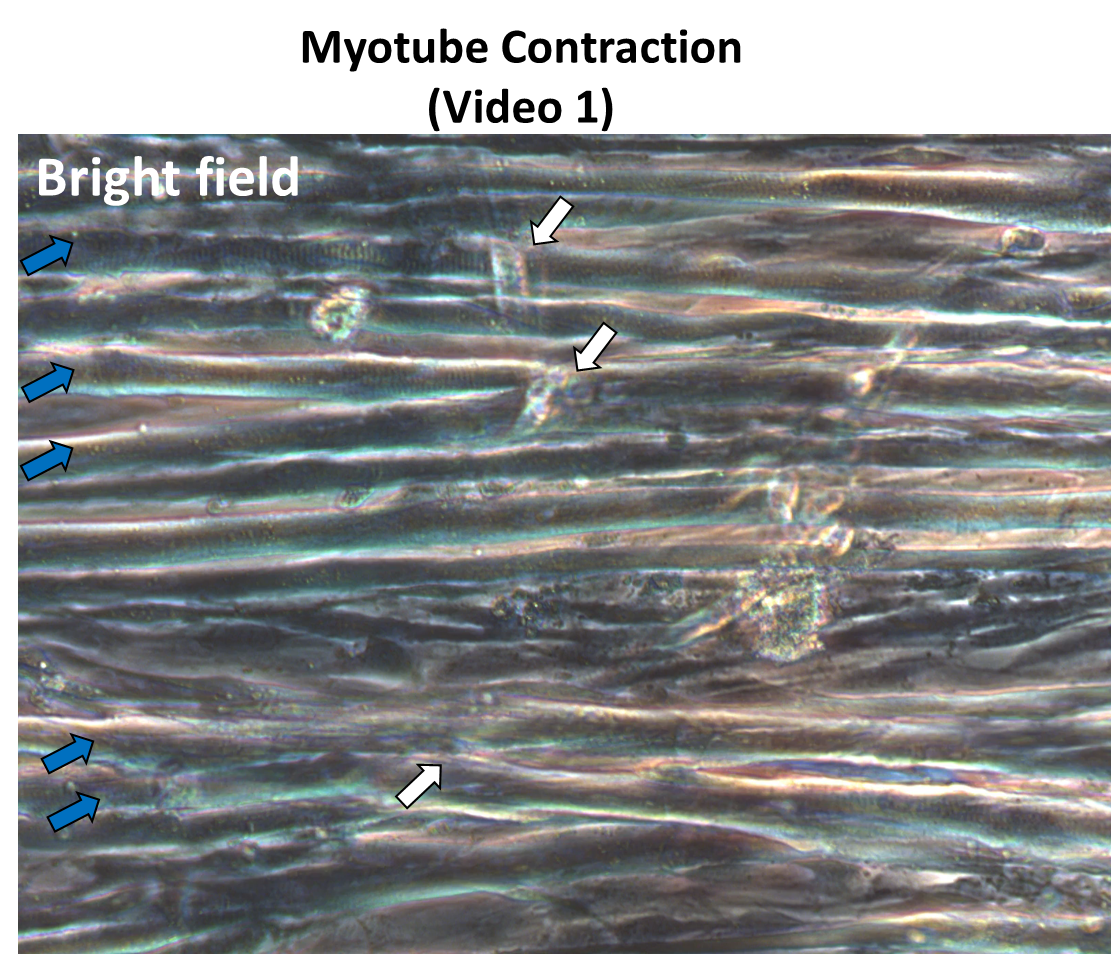


**Additional file 1: Fig 3. Relative positions of MNs and contracting myotubes for video 1.**

The live-cell image extracted from video 1 shows relative positions of MNs (white arrows) and contracting myotubes (blue arrows).
